# Novel Insights Into the Causal Effects and Shared Genetics Between Body Fat and Parkinson Disease

**DOI:** 10.1111/cns.70132

**Published:** 2024-11-22

**Authors:** Qian Zhao, Dongming Liu, Ancha Baranova, Hongbao Cao, Fuquan Zhang

**Affiliations:** ^1^ Department of Psychiatry The Affiliated Brain Hospital of Nanjing Medical University Nanjing Jiangsu China; ^2^ Department of Radiology Affiliated Drum Tower Hospital, Medical School of Nanjing University Nanjing Jiangsu China; ^3^ School of Systems Biology George Mason University Manassas Virginia USA; ^4^ Research Centre for Medical Genetics Moscow Russia; ^5^ Institute of Neuropsychiatry, The Affiliated Brain Hospital of Nanjing Medical University Nanjing Jiangsu China

**Keywords:** BMI, body fat, colocalization, cross‐trait meta‐analysis, Mendelian randomization, Parkinson disease

## Abstract

**Aims:**

Existing observational studies examining the effect of body fat on the risk of Parkinson disease (PD) have yielded inconsistent results. We aimed to investigate this causal relationship at the genetic level.

**Methods:**

We employed two‐sample Mendelian randomization (TSMR) to investigate the causal effects of body fat on PD, with multiple sex‐specific body fat measures being involved. We performed Bayesian colocalization analysis and cross‐trait meta‐analysis to reveal pleiotropic genomic loci shared between body mass index (BMI) and PD. Finally, we used the MAGMA tool to perform tissue enrichment analysis of the genome‐wide association study hits of BMI.

**Results:**

TSMR analysis suggests that except waist circumference, higher measures of body fatness are associated with a decreased risk of PD, including BMI (OR: 0.83), body fat percentage (OR: 0.69), body fat mass (OR: 0.77), and hip circumference (OR: 0.83). The observed effects were slightly more pronounced in females than males. Colocalization analysis highlighted two colocalized regions (chromosome 3p25.3 and chromosome 17p12) shared by BMI and PD and pointed to some genes as possible players, including *SRGAP3*, *MTMR14*, and *ADORA2B*. Cross‐trait meta‐analysis successfully identified 10 novel genomic loci, involving genes of *TOX3* and *MAP4K4*. Tissue enrichment analysis showed that BMI‐associated genetic variants were enriched in multiple brain tissues.

**Conclusions:**

We found that nonabdominal body fatness exerts a robust protective effect against PD. Our colocalization analysis and cross‐trait meta‐analysis identified pleiotropic genetic variation shared between BMI and PD, providing new clues for understanding the association between body fat and PD.

## Graphical abstract

Our two‐sample Mendelian randomization analysis suggested that except waist circumference, higher body mass index (BMI), body fat percentage, body fat mass, and hip circumference were protective against Parkinson disease (PD), and were more pronounced in females than in males. Colocalization analysis highlighted two colocalized regions shared by BMI and PD. Cross‐trait meta‐analysis successfully identified 10 novel genomic loci. They pointed to some genes as possible players, including *SRGAP3*, *MTMR14*, *ADORA2B*, *TOX3*, and *MAP4K4*. Enrichment analysis showed that BMI‐associated genetic variants were enriched in multiple brain tissues.

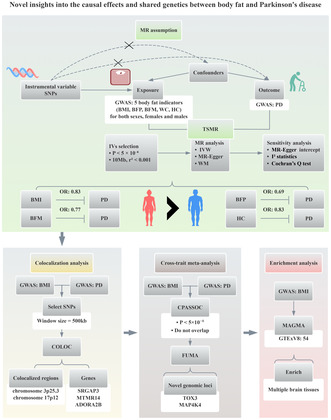

## 1 Introduction

Globally, the escalating issue of excessive weight gain impacts a staggering number of people [1]. Obesity is characterized by an accumulation of body fat, with an increase in several indicators, including body mass index (BMI), body fat percentage (BFP), whole body fat mass (BFM), waist circumference (WC), and hip circumference (HC) [2]. Across all age groups worldwide, sex disparities in obesity prevalence are noticeable, with 41% of females being obese compared to 38% of males [3]. Obesity is a multifaceted condition underlined by a variety of contributing factors, and compounded by many comorbidities, including cardiovascular diseases, Type 2 diabetes, arthritis, and even cancer [4]. For all these diseases, adiposity is a contributing factor. Notably, 30%~80% of the differences in human body weight are due to genetic factors. Additionally, dietary, behavioral, and social factors strongly contribute to the onset and the development of progressive weight gain, as well as, the success of weight management [5].

Parkinson disease (PD) ranks as the second most prevalent neurodegenerative condition, impacting over 6 million individuals globally [6]. PD is associated with profound disability and a diminished quality of life. It manifests with bradykinesia, resting tremor, stiffness, and postural instability, often accompanied by disturbances in nonmotor domains, encompassing cognitive, mental, and autonomic functions [7, 8]. An expanding body of experimental and clinical evidence supports the idea that the pathophysiological patterns of PD differ between males and females [9, 10]. The etiology of PD is composite and includes aspects of normal aging, genetic predisposition, and environmental exposures [11]. Inherited genetic variants are estimated to contribute approximately 25% to the overall risk of developing PD [12].

Both an increase in body fatness and PD are commonly described as public health concerns. Several epidemiological studies have suggested that general body fatness, with WC as a proxy, may contribute to PD [13]. However, in a nested case–control study, the BMI was inversely associated with PD, and the risks of PD risk were declining with the increment in BMI [14]. In another large cohort study of females with a substantial follow‐up, obesity was associated with a lower risk of PD, with a similar pattern found for WC when measured 20 years before diagnosis [15]. There is growing evidence that the relationship between obesity and mortality may change with age [16]. Data from a 25‐year‐long follow‐up showed that low BMI was associated with higher mortality among Japanese older adults [17]. Two previous Mendelian randomization (MR) studies also reported an inverse association of BMI with PD [18, 19].

It is important to note that studies of fatness‐related anthropometric parameters other than BMI are scarce. To our knowledge, no studies investigated the relationships between indicators of body fat such as percentage of arm fat, percentage of leg fat, and trunk fat mass with the risks of PD. This is surprising, given that specific and progressive losses of both visceral and subcutaneous fat, but not the muscle mass have been noticed in PD patients in several studies [20, 21], with an onset of lipopenia noticeable a few years before diagnosis [22].

Importantly, gender cohort analyses point out that the impacts of abdominal body fat on PD risk may depend on sex [13]. These observations prompt us to consider whether causal impacts of body fat indicators on the risks of PD risk vary in males and females, and whether the measures of fatness as such should serve as better predictors for the risk of PD than commonly utilized indicators of BMI. To this end, we explored the GWAS datasets, which have become a powerful tool for studying the genetic components of various human traits, including general body fatness and PD [4, 23], with the technique of MR capable of uncovering the causal relationship between the two conditions [24]. MR allows estimating the strength of the association between any given exposure and its outcome with the aid of genetic variables, which do not change over the life course, therefore, revealed associations with various outcomes are truly causal [25]. Since gamete formation follows the Mendelian law of inheritance stating that parental alleles are randomly assigned to each offspring, genetic variation is not affected by traditional confounding factors [26]. Colocalization analysis is a method used to study the association between genetic variants and phenotypes (or diseases) [27]. It is particularly useful to test whether the signals observed in two different genetic association studies are derived from the same genetic variants. Cross‐trait meta‐analysis is a method for the joint analysis of multiple phenotypes within a cohort to improve the statistical ability to detect genetic linkages and associations [28]. We used colocalization analysis and cross‐trait meta‐analysis to identify relevant genes between BMI and PD.

## 2 Methods

### 2.1 Study Design and Data Sources

The GWAS summary results used for this analysis were all from publicly available data (Tables S1–S4.). GWAS summary dataset of body fat characteristics from the Zenodo database included BMI, BFP, BFM, WC, and HC for males, females, and entire cohorts (https://zenodo.org/doi/10.5281/zenodo.8011558). The GWAS summary results for PD were from the study published in 2019 (*N* = 482,730) [23]. All participants were of European descent. Ethical approval was obtained in all original studies.

### 2.2 Two‐Sample MR Analysis

In R (version 4.0.5), we performed the two‐sample MR analysis (TSMR) with multiple measures of human body fatness as an exposure and PD as an outcome. The analysis employed three complementary methods integrated into TwoSampleMR (version 0.5.6) [29], including inverse variance weighted (IVW), weighted median, and MR‐Egger. These methods have distinct assumptions regarding horizontal pleiotropy. The IVW model served as our primary TSMR approach [30], assuming zero intercepts and yielding consistent causality estimates through fixed‐effects meta‐analysis. The MR‐Egger model assumes that pleiotropic effects are independent and applies weighted linear regression of outcome coefficients to exposure coefficients. Using intercepts of MR‐Egger regression to assess horizontal pleiotropy [30]. When the MR‐Egger intercepts are significantly different from zero, IVs lose their effectiveness. The heterogeneities were gauged by both *I* ^2^ statistics and Cochran's *Q* test (both *I* ^2^ > 0.25 and *p* < 0.05) [31]. Significant associations were determined based on the false discovery rate (FDR), computed from *p* values obtained in the IVW model. An IVW‐based FDR < 0.05 determined a significant correlation between the different indicators of body fatness on PD.

In the TSMR analysis, single‐nucleotide polymorphisms (SNPs) with genome‐wide significance (*p* < 5 × 10^−8^) were selected as IVs and further pruned using a clumping *r* ^2^ cut‐off of 0.001 within a 10 Mb window, using the 1000 Genomes Project Phase 3 (EUR). When performing TSMR analysis, we deleted the SNPs that did not exist in the outcome dataset and palindromic SNPs with intermediate allele frequencies. We reconcile each pair of exposure and outcome datasets by aligning the effect alleles of exposure and outcome.

### 2.3 Colocalization Analysis

We applied Bayesian colocalization analysis to assess colocalization between BMI and PD. We conducted the statistical analysis using R and the “coloc” package (version 5.2.3) [32]. The type of colocalization we applied was GWAS–GWAS, based on GWAS summary data obtained for BMI and PD. There are five assumptions in this approach: H0: no association with either trait; H1: association with Trait 1, not with Trait 2; H2: association with Trait 2, not with Trait 1; H3: association with Trait 1 and Trait 2, two independent SNPs; and H4: association with Trait 1 and Trait 2, one shared SNP. We chose a range of 250 kb up and down each SNP to divide the colocalization area, and defined colocalization based on high posterior probability (PP.H4.abf > 0.70).

### 2.4 Cross‐Trait Meta‐Analysis

We conducted a cross‐trait meta‐analysis to further identify pleiotropic loci affecting BMI and PD, using the CPASSOC package [28]. CPASSOC can integrate evidence of association for multiple, sometimes seemingly disparate phenotypes. When pleiotropic effects are present, this meta‐analysis significantly improves the statistical ability to detect genetic effects. CPASSOC provides two statistics, SHom and SHet. CPASSOC is performed using aggregated statistics from GWAS [33]. Pleiotropic SNPs shared between BMI and PD were identified at a significance level of *p* < 5 × 10^−8^. When the SNP does not overlap with any genome‐wide significant SNPs in the two previously reported GWAS traits, it is considered a new shared SNP. FUMA (https://fuma.ctglab.nl/) computes linkage disequilibrium (LD) structure, annotates the functions to SNPs, and maps them to genes. Using FUMA, we annotated the results of a meta‐analysis of BMI and PD [34].

### 2.5 Enrichment Analysis

We used the Multi‐Marker Analysis of GenoMic Annotation (MAGMA) tool in the FUMA online platform to perform tissue expression enrichment analysis of summary statistics of GWAS results of BMI [34, 35]. MAGMA analysis is a fast and flexible method for gene and generalized gene‐set analysis. This analysis was performed on 54 tissue types from the Genotype‐Tissue Expression Project (GTEx V8) [36].

## 3 Results

### 3.1 Two‐Sample MR Analysis

**FIGURE 1:**
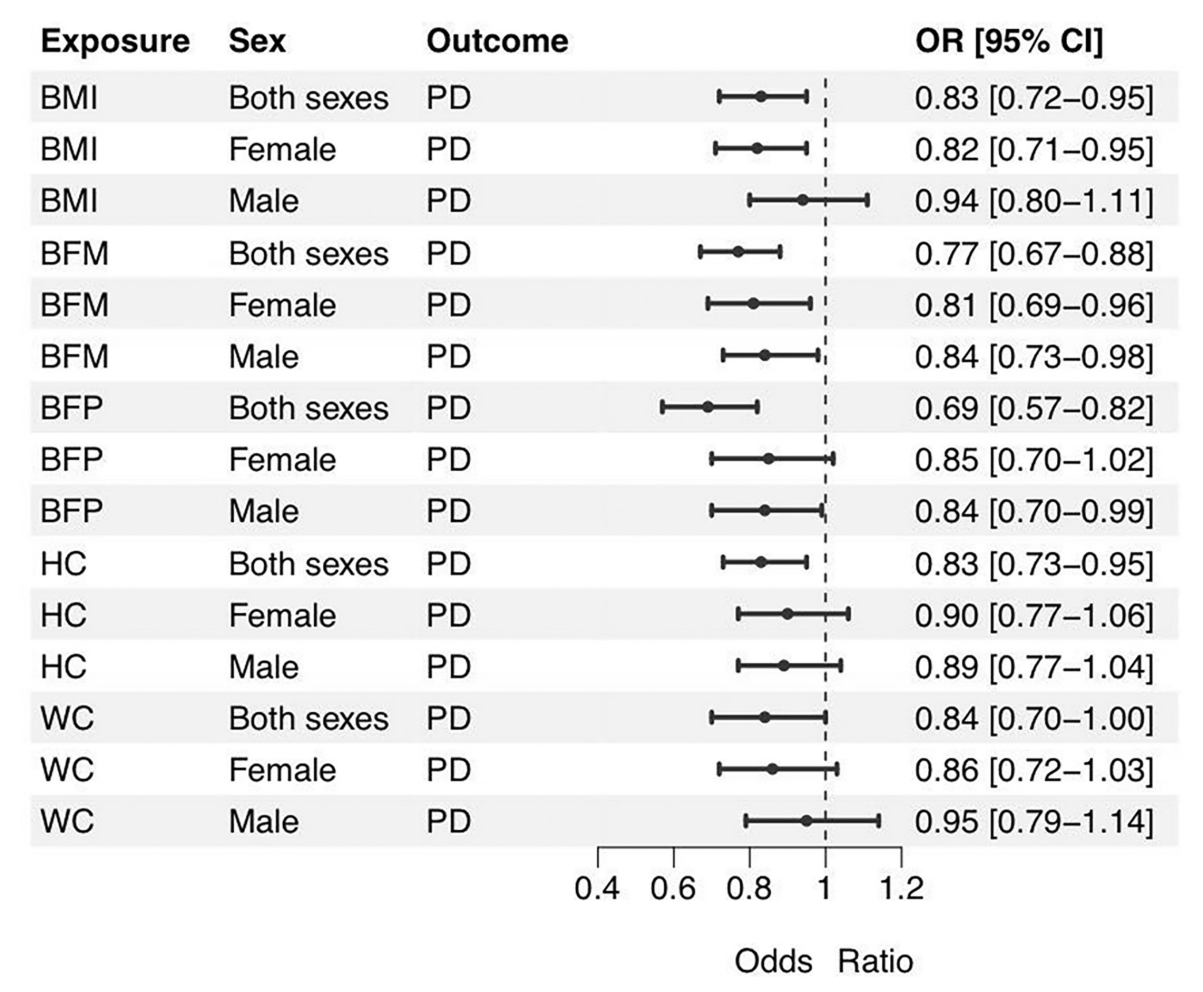
Forest plot of causal effects of body fat indicators on PD. BFM, Whole body fat mass; BFP, body fat percentage; BMI, body mass index; CI, confidence interval; HC, hip circumference; OR, odds ratio; PD, Parkinson disease; WC, waist circumference.

**FIGURE 2:**
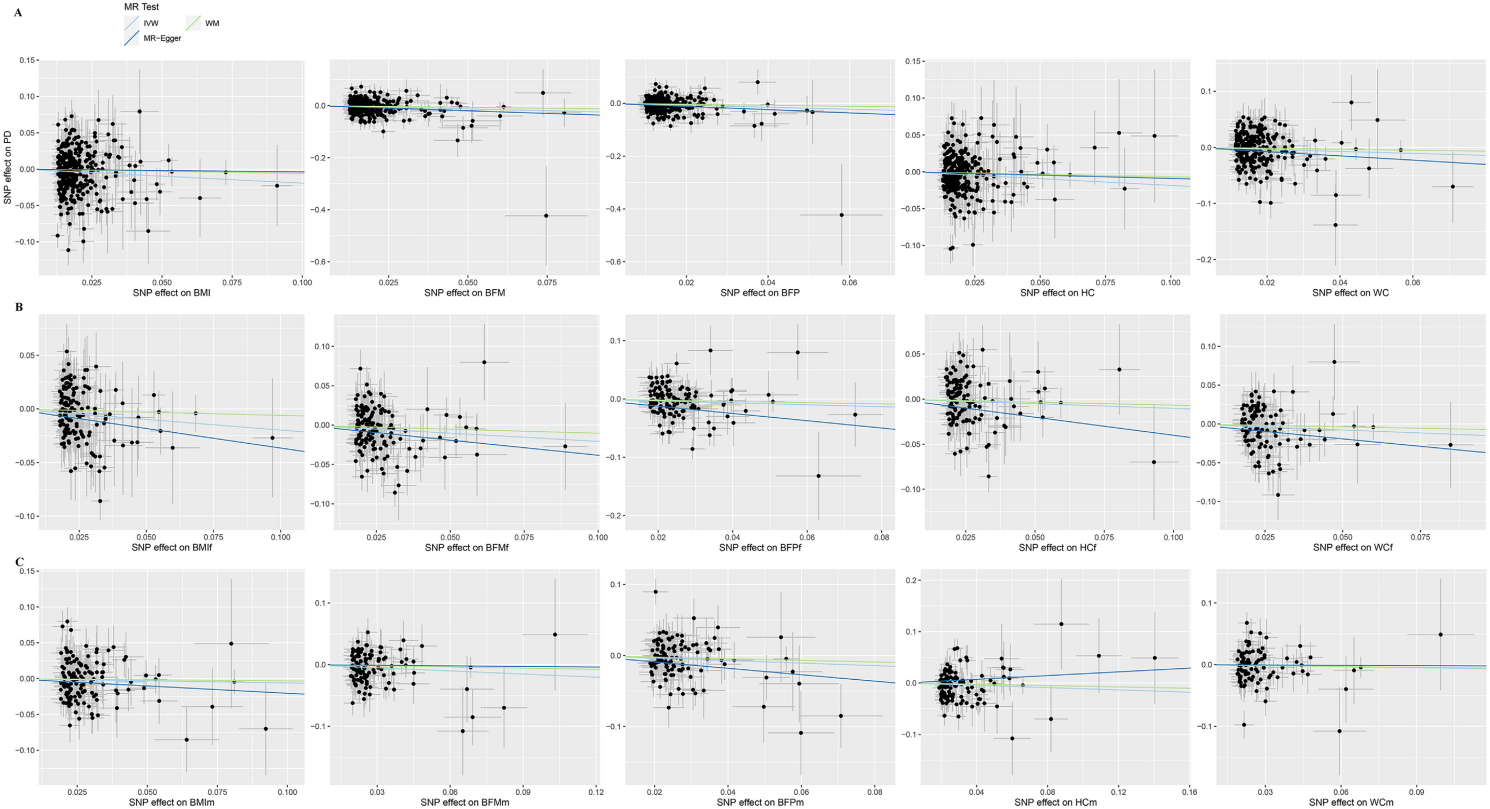
Scatter plot of causal effects of body fat measures on PD. (A) Both sexes. (B) Female. (C) Male. BFM, Whole body fat mass; BFP, body fat percentage; BMI, body mass index; HC, hip circumference; IVW, inverse variance weighted; PD, Parkinson disease; WC, waist circumference; WM, weighted median.

We performed the TSMR analysis aimed to investigate the causal effect of body fat on PD in the entire cohort, and also in male and female subcohorts, separately. Significant gender‐specific differences in the effects of various body fat indicators on the risks of PD were detected (Table 1, Figure 1, and Figure 2). Non–sex‐specific TSMR analysis showed that the genetic liability to higher BMI (OR: 0.83, 95% CI: 0.72–0.95, *p* = 8.37E‐03, FDR = 0.025), BFP (OR: 0.69, 95% CI: 0.57–0.82, *p* = 3.74E‐05, FDR = 5.61E‐04), BFM (OR: 0.77, 95% CI: 0.67–0.88, *p* = 1.22E‐04, FDR = 9.17E‐04), and HC (OR: 0.83, 95% CI: 0.73–0.95, *p* = 5.55E‐03, FDR = 0.025) was associated with reduced risk of PD. In females, these risks were negatively influenced by BMI (OR: 0.82, 95% CI: 0.71–0.95, *p* = 8.26E‐03, FDR = 0.025) and BFM (OR: 0.81, 95% CI: 0.69–0.96, *p* = 0.012, FDR = 0.030). In males, associations of BFP and BFM with reduced risk of PD were nominal. WC had no causal association with PD either in entire population (OR: 0.84, 95% CI: 0.70–1.00, *p* = 0.050, FDR = 0.083) or in males (OR: 0.95, 95% CI: 0.79–1.14, *p* = 0.552, FDR = 0.552) and in females (OR: 0.86, 95% CI: 0.72–1.03, *p* = 0.091, FDR = 0.124) analyzed separately.

**TABLE 1:** Causal effects of body fat indicators on PD.

Exposure	Sex	Outcome	*N*_IV	*b* (SE)	OR [95% CI]	*p*	FDR
BMI	Both sexes	PD	331	−0.192 (0.073)	0.83 [0.72–0.95]	8.37E‐03	0.025
BMI	Female	PD	141	−0.197 (0.075)	0.82 [0.71–0.95]	8.26E‐03	0.025
BMI	Male	PD	126	−0.061 (0.083)	0.94 [0.80–1.11]	0.461	0.494
BFM	Both sexes	PD	309	−0.261 (0.068)	0.77 [0.67–0.88]	1.22E‐04	9.17E‐04
BFM	Female	PD	138	−0.206 (0.082)	0.81 [0.69–0.96]	0.012	0.030
BFM	Male	PD	111	−0.169 (0.075)	0.84 [0.73–0.98]	0.024	0.052
BFP	Both sexes	PD	287	−0.377 (0.092)	0.69 [0.57–0.82]	3.74E‐05	5.61E‐04
BFP	Female	PD	118	−0.168 (0.093)	0.85 [0.70–1.02]	0.072	0.108
BFP	Male	PD	111	−0.179 (0.088)	0.84 [0.70–0.99]	0.043	0.080
HC	Both sexes	PD	303	−0.185 (0.067)	0.83 [0.73–0.95]	5.55E‐03	0.025
HC	Female	PD	125	−0.102 (0.082)	0.90 [0.77–1.06]	0.214	0.247
HC	Male	PD	126	−0.112 (0.075)	0.89 [0.77–1.04]	0.137	0.171
WC	Both sexes	PD	258	−0.177 (0.090)	0.84 [0.70–1.00]	0.050	0.083
WC	Female	PD	105	−0.155 (0.092)	0.86 [0.72–1.03]	0.091	0.124
WC	Male	PD	92	−0.056 (0.094)	0.95 [0.79–1.14]	0.552	0.552

Abbreviations: *b*, Effect size; BFM, whole body fat mass; BFP, body fat percentage; BMI, body mass index; CI, confidence interval; FDR, false discovery rate; HC, hip circumference; *N*_IV, number of instrumental variables; OR, odds ratio; PD, Parkinson disease; SE, standard error; WC, waist circumference.

In sensitivity analysis across the set of applied techniques, the directions of causal‐effect estimates revealed were largely the same (Table S2). No horizontal pleiotropy was detected in the result of the MR‐Egger model (P_pleiotropy > 0.05 and MR‐Egger intercept < 0.01). On the other hand, Cochran's *Q* test and the *I* ^2^ statistics suggested the heterogeneity of the partial‐effect estimates. Among the six significant causal effects identified, the protective effects of BMI, BFM, and HC on PD in both sexes and the protective effect of BFM on PD in females showed heterogeneity. Therefore, these results should be interpreted with caution.

### 3.2 Colocalization Analysis

**FIGURE 3:**
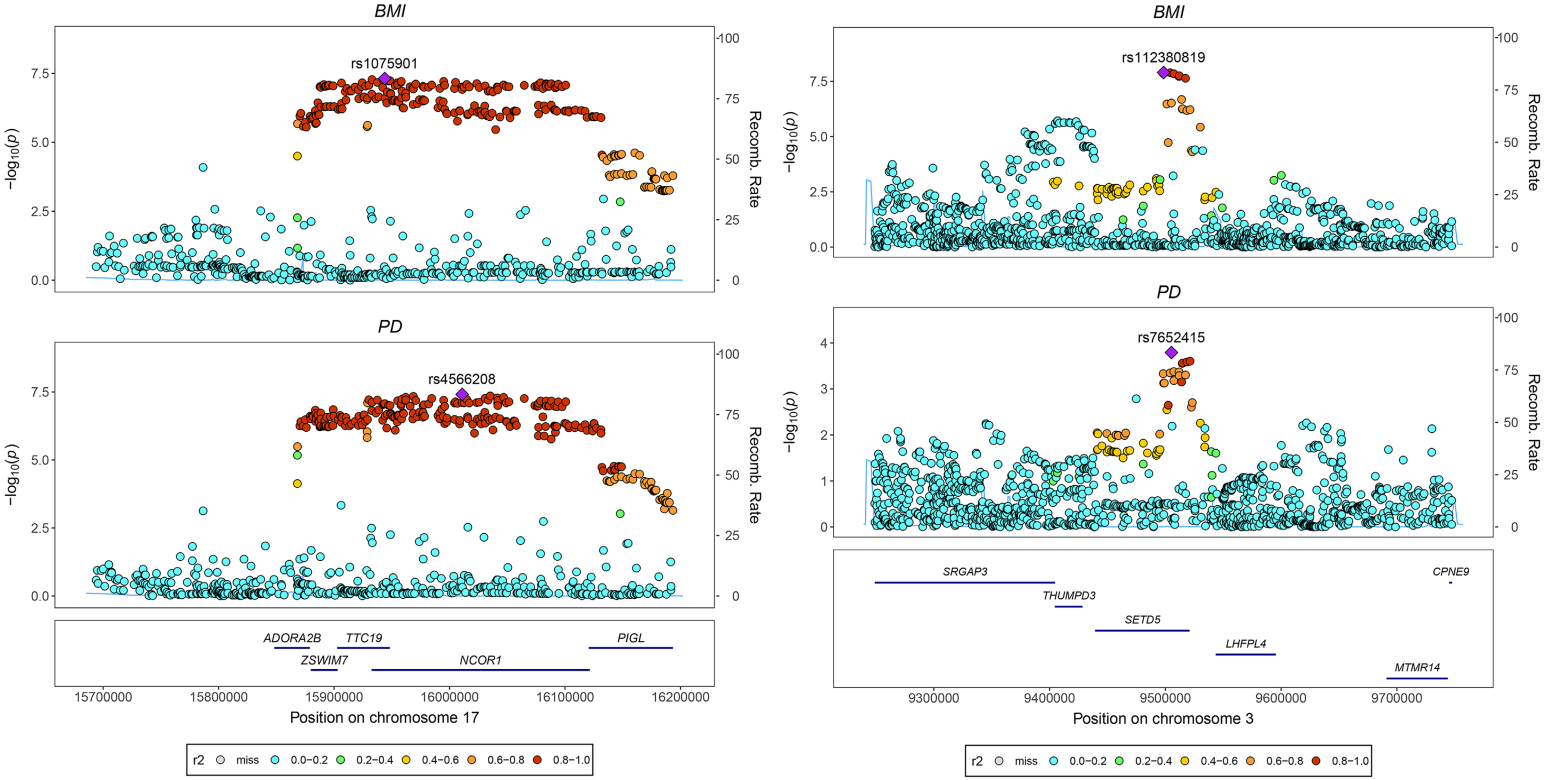
Two colocalized regions between BMI and PD. The *x*‐axis represents the genomic coordinates and the *y*‐axis represents the negative log_10_ transform *p* value for each genetic variant. BMI, Body mass index; PD, Parkinson disease.

In the paired GWAS of BMI and PD, two chromosomal regions were identified by colocalization analysis, with their top lead SNPs located on chromosome 3p25.3 and chromosome 17p12 (Table S3). Eleven genes were identified in the two colocalized chromosomal regions. They were *SRGAP3*, *THUMPD3*, *SETD5*, *LHFPL4*, *MTMR14*, and *CPNE9* on Chromosome 3, and *ADORA2B*, *ZSWIM7*, *TTC19*, *NCOR1*, and *PIGL* on Chromosome 17 (Figure 3).

### 3.3 Cross‐Trait Meta‐Analysis

**FIGURE 4:**
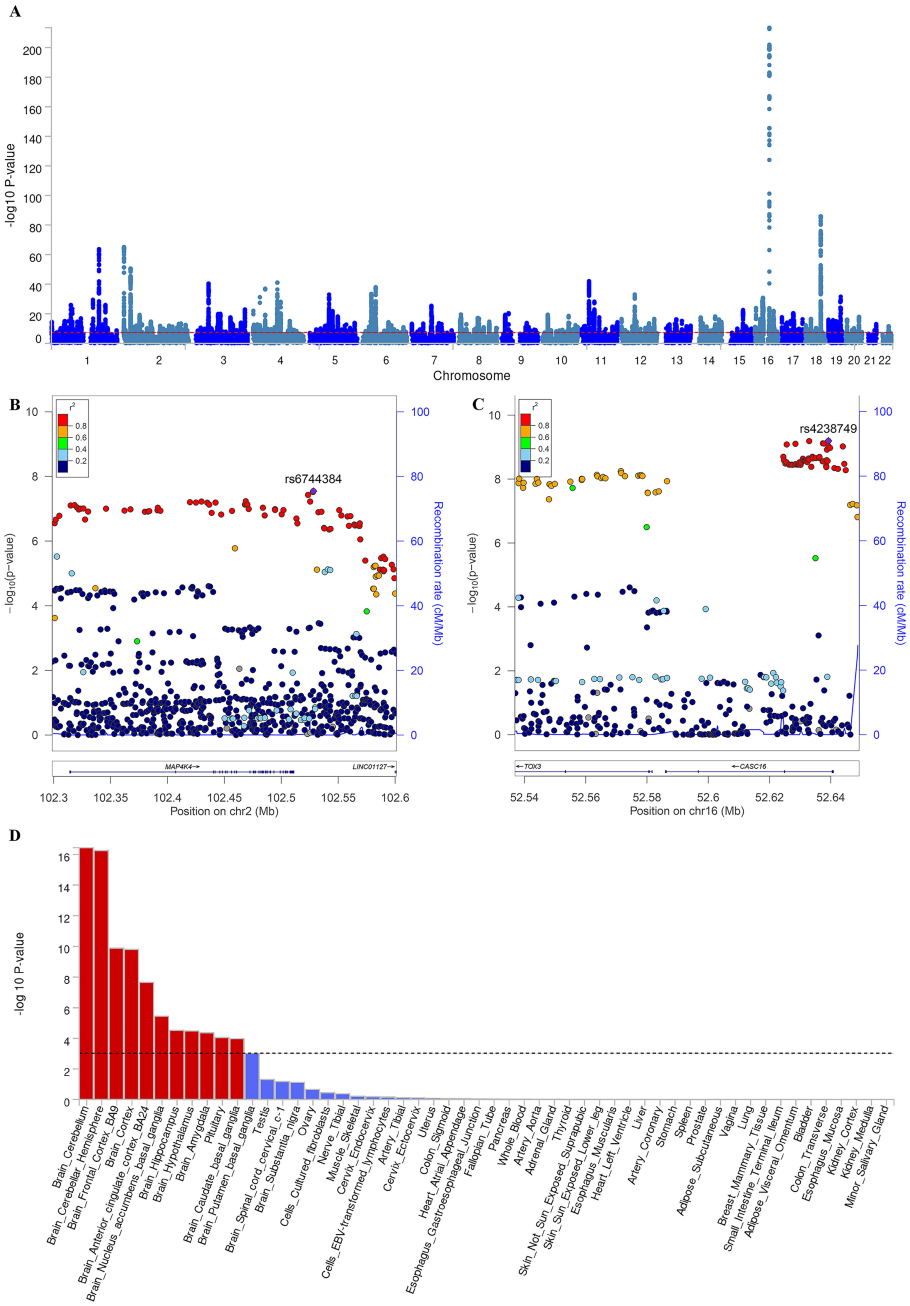
Cross‐trait meta‐analysis and enrichment analysis. (A) Manhattan plot of FUMA analysis of BMI and PD. The *x*‐axis is the chromosomal position of SNPs, and the *y*‐axis is the significance of the SNPs (−log_10_P). (B, C). The genomic loci of BMI and PD. (D) Enrichment analysis of BMI.

The cross‐trait meta‐analysis detected 395 shared risk SNPs between BMI and PD (Table S4). The most significant SNP in our analysis was rs11642015 (*p* = 3.54E‐214) located at 16q12.2 (Table S4, and Figure 4A). Ten of these SNPs were newly identified. These 10 SNPs were mainly located at genomic regions 2p23.3 (harboring *SNX17* and *GCKR*), 2q11.2 (harboring *MAP4K4* and *IL1R2*), 3p14.3 (harboring *ABHD6*), 5q21.1 (harboring *RN7SL802P*), 12q14.2 (harboring *TMEM5*, *TMEM5‐AS1*, *PABPC1P4*, and *SRGAP1*), 12q21.1 (harboring *RAB21*), 12q24.31 (harboring *FAM101A*), 14q22.3 (harboring *KTN1‐AS1* and *KTN1*), and 16q12.1 (harboring *TOX3* and *CASC16*) (Table 2 and Figure 4B,C). The quantile–quantile plots showed the distribution of observed versus expected values of the meta‐analyzed statistics on the –log_10_(P) scale under the unrelated null model (Figure S1).

**TABLE 2:** Novel loci identified in the cross‐trait meta‐analysis of BMI and PD.

SNP	CHR	BP	Start:End	A1/A2	*p*	Genes
rs1260326	2	27730940	27598097:27752871	C/T	4.48E‐08	SNX17; GCKR
rs6744384	2	102528292	102298722:102601271	A/G	2.85E‐08	MAP4K4; IL1R2
rs4488803	3	58218352	58218352:58225974	A/G	4.66E‐11	ABHD6
rs12521888	5	100701554	100595773:100925684	A/T	2.46E‐08	RN7SL802P
rs2362796	12	61287551	61022426:61305005	C/T	4.64E‐08	
rs111248163	12	64225791	64086743:64310719	G/T	4.76E‐08	TMEM5; TMEM5‐AS1; PABPC1P4; SRGAP1
rs61754230	12	72179446	72179446:72179446	C/T	2.09E‐10	RAB21
rs7956959	12	124515574	124503803:124576837	C/T	9.94E‐09	FAM101A
rs8007832	14	56035579	55998651:56166871	C/T	3.94E‐08	KTN1‐AS1; KTN1
rs4238749	16	52639234	52536885:52649310	A/C	6.35E‐10	TOX3; CASC16

Abbreviations: BP, base pair; CHR, chromosome; SNP, single nucleotide polymorphisms.

### 3.4 Enrichment Analysis

BMI‐based GWAS results show that the brain and pituitary were the tissues with the highest enrichment (Figure 4D). There are 11 subtype‐specific tissue types involved, including pituitary and 10 brain subregions (cerebellum, cerebellar hemisphere, frontal cortex BA9, cortex, anterior cingulate cortex BA24, nucleus accumbens basal ganglia, hippocampus, hypothalamus, amygdala, and caudate basal ganglia).

## 4 Discussion

Association between central obesity, as measured by WC, and the subsequent development of PD has been investigated in several studies, which have pointed at insulin resistance as a key modulator in the link between them [37, 38]. On the other hand, not every individual overweight or obese is insulin resistant, pointing to the possibility that the effects of body fatness traits and carbohydrate metabolism impairment may be independent or even counterbalancing each other, which may explain mixed findings of non–WC‐based studies of the association between the lifetime changes in body mass and the development of PD. When analyzing the relationships between characteristics of body fatness with PD, we found causal protective effects of the accumulation of fat in the body.

In this, the results of our study align with previous functional imaging observations pointing that patients with a higher BMI may experience neuroprotective benefits, particularly in terms of preserving cognitive function and neural networks [39]. At least in part, these benefits may be explained by the elevation of the levels of insulin‐like growth factor II, which are proportional to weight gain in humans [40] and neuroprotective in a mouse model of PD with synuclein‐alpha accumulation [41].

Moreover, subsequent colocalization analysis has highlighted several genes within two colocalized chromosomal regions that may be involved in modulating the causal effects of BMI on PD. These genes, namely, *SRGAP3, THUMPD3, SETD5, LHFPL4, MTMR14*, and *CPNE9*, as well as *ADORA2B, ZSWIM7, TTC19, NCOR1*, and *PIGL*, are located in human chromosomes 3p25.3 and 17p12, respectively.

*ADORA2B* encodes one of the adenosine receptors with adenylate cyclase activity. Suggestively associated with PD within one of the previous GWAS, this gene has been investigated for its neuroprotective effects in the Drosophila model and showed alleviation of α‐synuclein–induced phenotypes [42]. On the other hand, methylated CpGs within the *ADORA2B* locus were shown to mediate an association between BMI and blood pressure [43], while some other SNPs in *ADORA2B* highly correlate with inflammatory biomarkers IL‐6 and C‐reactive protein and other obesity‐related traits [44].

*MTMR14*, a gene that encodes a myotubularin‐related protein, has been reported to inhibit deleterious overactivation of autophagy, which is frequently associated with various neurodegenerative diseases, including PD [45]. In addition, studies have shown that a deficiency of *MTMR14* may induce metabolic dysfunction and inflammation [46].

The product of *SRGAP3* suppresses the migration of cells and, therefore, plays a role in multiple neurodevelopmental processes [47]. Genomic locus with the *SRGAP3* gene has reached genome‐wide significance for an association with BFP in at least one previous GWAS [48]. *SRGAP3* facilitates the process of axon guidance and is a part of the PD candidate pathway regulating dopaminergic nerve cells [49].

Furthermore, our cross‐trait meta‐analysis newly identified 10 loci significantly associated with BMI and PD, involving 16 protein‐coding genes, such as *TOX3* and *MAP4K4*.

*TOX3* has been widely recognized to be associated with breast cancer, and it has been suggested that it may be a modulator of estrogen receptor‐mediated gene expression [50]. On average, women had a higher percentage of body fat. *TOX3* is expressed in some regions of the brain, including the substantia nigra and cortex. *TOX3* may act as a nerve cell survival factor through calcium‐mediated transcriptional induction [51]. However, in PD, women are less susceptible. Further research is needed to test the hypothesis that *TOX3* may be involved in the protective effect of BMI on PD through different effects on estrogen receptor‐mediated gene expression.

Differentially expressed *MAP4K4* in PD patients encodes a serine/threonine kinase whose activation has been shown to mediate motor neuron degeneration in amyotrophic lateral sclerosis [52, 53]. In addition, *MAP4K4* inhibits the activation of peroxisome proliferation‐activated receptors‐γ and adipogenesis. An experiment found that the higher the BMI of the donor, the lower the number of preadipocytes that differentiate into adipocytes in subcutaneous abdominal adipose tissue. The potential mechanism may be increased expression of *MAP4K4* in preadipocytes of obese individuals [54].

Another important finding reported here is that the protective effects of body fatness‐related characteristics were more evident in females. On average, percentages of body fat in males are lower, especially in older cohorts [55, 56]. The sex differences in the amount, location, and metabolism of adipose tissue are well described [57]. Sex‐related differences in PD indicate that males are twice as likely to develop PD as females [58]. A study has found that small‐worldness topology in gray matter covariance networks is significantly modified by sex in PD [59]. Furthermore, the magnitude of PD risk factors, motor and nonmotor symptoms, and response to treatment vary greatly between females and males [58]. Observed differences cannot be explained by demographics alone, with estrogen's effects on dopaminergic nerve cells and pathways in the brain [60] being commonly cited as a protective factor, which is certainly more pronounced in premenopausal females [61, 62, 63]. In postmenopause, both estrone and estradiol are produced in visceral adipose proportionally to the size of this tissue compartment. Additionally, subcutaneous fat produces estradiol. The relative size of these depots of fat influences overall estrogen exposure in postmenopausal females, pointing at possible mechanisms mediating protection against PD in a sex‐specific way [64]. Future research is warranted to further elucidate the functional relationship between body fatness and neurodegeneration.

In future studies, it should be interesting to dissect whether other negative associations previously seen as alleviating the risk of PD, including smoking [65] and caffeine consumption [66, 67], may be mediated by body fatness‐related characteristics. In the case of smoking, there is an opinion that tobacco smoking may aid in controlling body weight due to the appetite‐suppressive effect of nicotine. Nevertheless, MR‐based studies support the opposite notion of the positive causal relationship between smoking initiation and obesity‐related traits [68], possibly mediated by genes, contributing to the intensity of addiction. In a similarly designed study, genetically predicted coffee consumption was positively associated with both obesity and a higher waist‐to‐hip ratio [69]. Moreover, the protective effects of coffee are most pronounced in patients with mutations in *LRKK2* [70], a gene most recently identified as one of the hub regulators in obesity phenotypes [71], thus, indicating the potential involvement of specific molecular mechanisms.

Genetic findings have the potential to provide constructive improvement and innovative perspectives in the clinical setting. Here, we have used the largest existing PD dataset to elucidate the relationship between body fat and PD risk. Our study is less susceptible to confounding than traditional observational studies and is not affected by reverse causation because genes are constant and do not change with disease progression. We also conducted pleiotropic and sensitivity analyses to minimize potential biases. On the other hand, MR studies are prone to so‐called survival bias, and our study is not excluded from this rule. One should also consider that body fat distribution varies by ethnicity, for example, within the same BMI band, Asians will have the highest BFP than other ethnicities [72]. This study focused on individuals of European ancestry, thus, limiting the generalizability of our results.

## 5 Conclusion

The results of this TSMR analysis support that body fat has a protective effect on PD. Distinguishing between sexes, female body fat has a more significant negative causal effect on PD. In addition, the genes identified by colocalization analysis and cross‐trait meta‐analysis of BMI and PD have the potential to serve as new therapeutic targets for related diseases.

## Author Contributions

Q.Z.: writing – original draft; writing – review and editing; and visualization. D.L., A.B., and H.C.: writing – review and editing. F.Z.: conceptualization; formal analysis; and supervision. All authors contributed to the revision of the manuscript. All authors approved the final version.

## Conflicts of Interest

The authors declare no conflicts of interest.

## Supporting information


Figure S1.

Tables S1–S4.

## Data Availability

All data generated or analyzed during this study are included in this published article and its supplementary information files.
