# Supplementary material for: Novel Insights Into the Causal Effects and Shared Genetics Between Body Fat and Parkinson Disease
Source: CNS Neurosci Ther. 2024 Nov 22;30(11):e70132. doi: 10.1111/cns.70132 (PMC11584348; doi:10.1111/cns.70132)

**Supplementary Figure 1. The Quantile-Quantile plot of the meta-analysis.** The x-axis is the expected values on the -log10(P) scale, and the y-axis is the observed values on the -log10(P) scale.


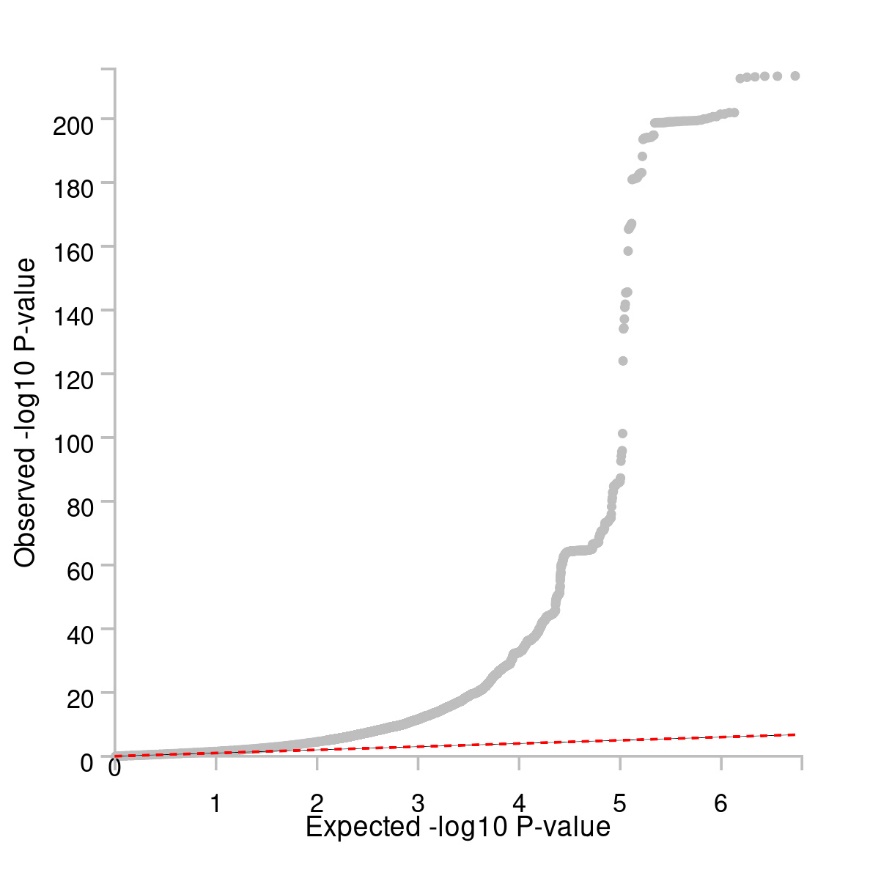

Supplement: Supplementary file 1 — Figure S1. [file CNS-30-e70132-s002.docx]
